# Chitinases and Chitinase-Like Proteins as Therapeutic Targets in Inflammatory Diseases, with a Special Focus on Inflammatory Bowel Diseases

**DOI:** 10.3390/ijms22136966

**Published:** 2021-06-28

**Authors:** Marzena Mazur, Anna Zielińska, Marcin M. Grzybowski, Jacek Olczak, Jakub Fichna

**Affiliations:** 1 Department of Biochemistry, Faculty of Medicine, Medical University of Lodz, 92-215 Lodz, Poland; m.mazur@oncoarendi.com (M.M.); ania.zielinska0122@gmail.com (A.Z.); 2 OncoArendi Therapeutics SA, 02-089 Warsaw, Poland; m.grzybowski@oncoarendi.com (M.M.G.); j.olczak@oncoarendi.com (J.O.)

**Keywords:** mammalian chitinase, inflammatory bowel diseases, YKL-40, chitinase inhibitor

## Abstract

Chitinases belong to the evolutionarily conserved glycosyl hydrolase family 18 (GH18). They catalyze degradation of chitin to *N*-acetylglucosamine by hydrolysis of the β-(1-4)-glycosidic bonds. Although mammals do not synthesize chitin, they possess two enzymatically active chitinases, i.e., chitotriosidase (CHIT1) and acidic mammalian chitinase (AMCase), as well as several chitinase-like proteins (YKL-40, YKL-39, oviductin, and stabilin-interacting protein). The latter lack enzymatic activity but still display oligosaccharides-binding ability. The physiologic functions of chitinases are still unclear, but they have been shown to be involved in the pathogenesis of various human fibrotic and inflammatory disorders, particularly those of the lung (idiopathic pulmonary fibrosis, chronic obstructive pulmonary disease, sarcoidosis, and asthma) and the gastrointestinal tract (inflammatory bowel diseases (IBDs) and colon cancer). In this review, we summarize the current knowledge about chitinases, particularly in IBDs, and demonstrate that chitinases can serve as prognostic biomarkers of disease progression. Moreover, we suggest that the inhibition of chitinase activity may be considered as a novel therapeutic strategy for the treatment of IBDs.

## 1. Introduction

Chitinases are widely distributed enzymes present in many organisms including insects, plants, bacteria, fungi, and mammals [1,2,3,4]. They catalyze degradation of chitin to *N*-acetylglucosamine by hydrolysis of the β-(1-4)-glycosidic bonds. Although mammals do not synthesize chitin, they possess two enzymatically active chitinases, i.e., chitotriosidase (CHIT1) and acidic mammalian chitinase (AMCase), as well as several chitinase-like proteins (CLPs; YKL-40, YKL-39, oviductin, and stabilin-interacting protein). In most mammals, e.g., rodents, chitinases protect against chitin-containing organisms that are either inhaled or ingested; however, human exposure to chitin is limited. Nevertheless, chitinases are highly upregulated in diseases related to chitin exposure such as sarcoidosis, nonalcoholic steatohepatitis (NASH) [5], Crohn’s disease (CD), and amyotrophic lateral sclerosis (ALS) [6] and in organs and tissues inaccessible to chitin-containing organisms. Therefore, in humans, chitinases and CLPs are believed to have evolved to perform various protective functions associated with inflammatory and fibrotic pathologies.

## 2. Chitinases and CLPs

### 2.1. CHIT1

The first chitinolytic enzyme discovered in humans is CHIT1, the gene of which is located on chromosome 1q31-q32. CHIT1 exists as two major isoforms with isoelectric points of 7.2 and 8.0 and molecular masses of 50 and 39 kDa, respectively [7]. The enzyme is expressed in both normal and pathological conditions, mostly by activated macrophages, but it is also present in other cell types, such as neutrophils, bronchial epithelial cells or Kupffer cells. CHIT1 has been implicated in the pathogenesis of multiple fibrotic lung diseases including idiopathic pulmonary fibrosis (IPF), sarcoidosis, chronic obstructive pulmonary disease (COPD), and asthma, as well as in other diseases with inflammatory or fibrotic phenotypes such as NASH, diabetic nephropathy, and ALS [5,6,8,9]. It is worth mentioning that some populations have complete enzyme deficiency due to homozygosity to the 24-bp duplication mutation at exon 10 of the CHIT1 gene [10,11,12].

### 2.2. AMCase

AMCase is another enzymatically active chitinase identified in humans. Unlike CHIT1, AMCase has an acidic pH optimum of around 2 [13]. The gene-encoding AMCase, also called chitinase acidic (CHIA) gene, is located on chromosome 1q13 and is highly expressed in the human stomach and at a lower level in the lungs as well as in human gastric mucosa [14]. As the enzyme is stable in the acidic environment, it is active in the gastric juice and may act as a defense against chitin-containing organisms [15]. Noteworthy, the expression level of AMCase in the stomach tissues is significantly higher in mice than in humans [16]. Although mouse and human AMCase have a similarity higher than 80%, these two proteins significantly differ in terms of their enzymatic activities. The data presented by Okawa et al. [17] show that the chitinolytic activity of human AMCase is significantly reduced as compared to the murine enzyme, i.e., the chitinolytic activities of the human AMCase are 1/75 and 1/11 of the activities of the mouse AMCase at pH 2.0 and 4.0, respectively. These important factors should be taken into careful consideration while using experimental animal models for the study of human diseases and the interpretation of the results. In animal models, AMCase was demonstrated to be induced during the Th2-type inflammation through an IL-13-dependent mechanism, and it has been implicated in the pathogenesis of asthma, as well as other diseases with the Th2 inflammatory phenotype, such as eosinophilic esophagitis or allergic ocular pathologies [18]. AMCase is highly expressed in the glandular cells of the stomach and intestinal tissues under normal physiological conditions, purportedly to aid in host defense and food processing.

### 2.3. CLPs

The most studied CLP is YKL-40 (chitinase-3-like protein 1 (CHI3L1)), a 40 kDa heparin- and chitin-binding glycoprotein which contains a highly conserved chitin-binding domain. It functionally lacks chitinase activity due to the mutation of catalytic glutamic acid (Glu140) into leucine (Leu119) [19,20]. The human protein is named YKL-40 based on its three *N*-terminal amino acids, i.e., tyrosine (Y), lysine (K), and leucine (L), and its molecular weight is 40 kDa. However, several other names are also in use: human cartilage glycoprotein-39 (HC-gp39) [21], 38 kDa heparin-binding glycoprotein (Gp38k) [22], chondrex [23], and 40 kDa mammary gland protein (MGP-40) [24]. YKL-40 has the ability to bind certain proteins and carbohydrates. In addition to chitin, several physiological ligands have been identified, e.g., heparin, chitohexaose, cellohexaose, hyaluronic acid, and collagen [25]. A recent study showed that the functional domain responsible for heparin binding and the putative biological function of YKL-40 is a domain containing arginine and lysine (residues 334–345) at the C-terminus of the protein [26]. YKL-40 is expressed by a multitude of cell types including macrophages, neutrophils, synoviocytes, chondrocytes, fibroblast-like cells, smooth muscle cells, endothelial cells, and cancer cells. Moreover, YKL-40 is secreted in vitro by numerous human cancer cell lines of various origins, including glioblastoma, ovarian cancer, prostate cancer, osteosarcoma, and malignant melanoma [27,28,29,30], and the elevated serum level of YKL-40 is suggested to be an indicator and reliable biomarker of poor prognosis in advanced cancer patients. It was also shown that YKL-40 expression is upregulated in colon cancer cells and has a significant correlation with macrophage infiltration and microvessel density in the tumors of colorectal cancer patients and in a xenograft mouse model [31]. However, the overall biological function of YKL-40 in human cancers still remains unknown.

YKL-40 expression is also found in tissues undergoing inflammation and intensive re-modelling. For example, it is not synthesized by healthy human chondrocytes in vivo; however, mRNA for YKL-40 is detected in osteoarthritic cartilage [21,32]. Immunohistochemical analysis showed that YKL-40 is expressed in chondrocytes of osteoarthritic cartilage [33]. The group of Mizoguchi et al. [34,35] has identified that YKL-40 is actively produced by colonic epithelial cells and lamina propria macrophages under inflammatory conditions. They also demonstrated an unexpected role for YKL-40 in enhancing bacterial adhesion and invasion into colonic epithelial cells.

Another CLP family member—YKL-39 (chitinase 3-like protein 2 (CHI3L2))—is a 39 kDa protein present in humans but absent in rodents. It is produced by articular chondrocytes and synoviocytes as well as in the lung and heart [36]. YKL-39 expression is increased in osteoarthritic articular chondrocytes and can serve as a marker for arthritic joint disease [37]. YKL-39 is also identified in tumor-associated macrophages in human breast cancer as a proangiogenic factor [38].

The only protein of the human GH18 possessing an *O*-glycosylated mucin-like region as well as a chitinase-like domain is oviductin (mucin 9 (MUC9); oviduct-specific glycoprotein (OGP)). It is exclusively expressed and secreted by the oviductal epithelium in time of ovulation, which would suggest its regulatory role during fertilization [39,40,41]. However, the studies performed in oviductin knockout mice did not confirm that [42]. The specific function of this protein in humans is thus to be established.

Kzhyshkowska et al. [43] revealed that human macrophages produce another catalytically inactive protein, known as stabilin-1 or interacting chitinase-like protein (SI-CLP). SI-CLP belongs to the GH18 family and interacts with the endocytic/sorting receptor stabilin-1. In vivo, high amounts of SI-CLP are detected in macrophages from bronchoalveolar lavage of patients with chronic airway inflammation as well as in Raji cells, Jurkat cells, various tumor cell lines, and CD3+ T cells isolated from peripheral blood of healthy donors.

## 3. Overview of the Role and Significance of Chitinases in Human Diseases

The natural substrate for chitinases is chitin—one of the most abundant polysaccharides that is found in nature as a component of fungal cell walls, the shells of crustaceans, and the exoskeletons of arthropods including house dust mites [44,45]. Besides its structural role, this biopolymer is also an important nutrient for lower life forms such as bacteria [46]. The widespread environmental presence of chitin and chitinolytic enzymes implies constant exposure to chitin, through the mucosa of both the respiratory and digestive systems [47,48]. This is reflected in the variety of diseases, in which chitin and chitinolytic enzymes appear to play significant functions. However, how these enzymes function has not been fully understood.

### 3.1. Inflammatory Lung Disorders

Asthma is an airway inflammation, in which eosinophils infiltrate the lung tissue leading to mucus overproduction, airway obstruction, and hyper-responsiveness [49]. Exaggerated Th-2 involvement leads to the induction and maintenance of the inflammation [49,50]. In animal models of ovalbumin-induced asthma, AMCase is overexpressed, and administration of anti-AMCase antibody or allosamidin, demethylallosamidin (chitinase inhibitors) or bisdionin F (an AMCase-selective inhibitor) leads to alleviation of asthma symptoms and a decrease in Th-2 inflammation, tissue eosinophilia, and leukocyte accumulation [51,52]. On the other hand, some studies show that in human lungs AMCase is mostly inactive and that AMCase-deficient mice present spontaneous pulmonary fibrosis with the accumulation of chitin polymers [48,51]. AMCase-deficient epithelial cells express fibrosis-associated gene sets linked with cell stress pathways. Mice with lung fibrosis due to the telomere dysfunction and humans with interstitial lung disease also accumulate excess chitin polymers in their airways [48]. Apart from AMCase, increased levels of YKL-40 are also described in asthma patients [49].

High levels of YKL-40, as well as high numbers of YKL-40-producing macrophages and epithelial cells, are present in the lungs of patients suffering from IPF [49,53]. Lung fibrosis is significantly reduced in CHIT1 knockout mice in a bleomycin-induced lung fibrosis animal model, and it is suggested that this chitinase plays an important role in tissue remodeling and fibrogenesis in the lung [54].

Sarcoidosis is a multisystemic disease, characterized by the formation of noncaseating granulomas. Activated, proliferating T cells and mononuclear phagocytes accumulate in various areas, such as the lung, lymph nodes, eyes, skin, liver, kidneys, muscles joints, and the central nervous system, leading to granuloma formation [55]. Sarcoidosis patients have higher CHIT1 serum concentrations than healthy individuals [8,56]; its activity in bronchoalveolar lavage (BAL) and serum is also significantly increased, especially in the advanced stages of the disease. YKL-40 has also been found to be associated with pulmonary sarcoidosis. It is expressed by macrophages and giant cells in pulmonary sarcoid granulomas, and its serum levels reflect the disease activity and fibrosis progression [57].

### 3.2. Neuroinflammation

Alzheimer’s disease (AD) is a degenerative disorder of the nervous system, in which senile plaques are formed. They are built of amyloid, degenerated neurons, and insoluble beta-amyloid fibril deposits. Pathophysiology of ischemic cerebrovascular dementia (CvD) includes cerebral ischemia and cognitive deterioration [57]. Both entities are caused partially by inflammatory processes and T cell immunity. Increased expression levels of CHIT1 mRNA are observed in AD and CvD [58]. Moreover, the CHIT1 activity and the YKL-40 level are markedly increased in cerebrospinal fluid of AD patients [49]. Finally, CHIT1 has become a marker specific for macrophage activation during the stroke, and its activity correlates with the grade of the stroke [57].

ALS is a neurodegenerative disease characterized by the degeneration of both upper and lower motor neurons, which leads to muscle weakness and eventually death from respiratory failure [59]. CHIT1 expression levels are increased in microglia and macrophages in the spinal cord and cerebrospinal fluid levels of ALS patients. CHIT1 expression correlates with disease severity and progression [6,60]. Administration of CHIT1 increased microglial numbers and astrogliosis in the ventral horn with a concomitant increase in the levels of proinflammatory cytokines. It also results in the reduction of motor neurons [60]. YKL-40 and YKL-39 mRNA levels are increased in the motor cortex in ALS and correlate with disease progression [61].

### 3.3. Metabolic Diseases

Atherosclerosis is an inflammatory disease of the arterial wall, in which the lipids and fibrous matrix progressively accumulate. Macrophages infiltrating the vessel wall accumulate lipids becoming lipid-laden “foam cells”. Chronic atherogenesis leads to thickening of tunica intima, narrowing of the vessel’s lumen and thrombosis [51]. Increased expression of YKL-40 mRNA is found in macrophages in early sclerotic lesions and in the macrophages that infiltrate deep into the lesion [54]. CHIT1 activity in macrophages is thus acknowledged as a biomarker of plaque development [51]. Serum levels of CHIT1 are also elevated (up to 55-fold) when compared to in healthy controls. Moreover, the CHIT1 activity level in serum is related to the severity of the atherosclerotic lesions and the age of the probands.

Diabetes mellitus type 2 (DM2) is characterized by hyperglycemia, insulin resistance and inadequate compensatory insulin excretion, leading to chronic structural and functional destruction of tissues and organs. Ischemic microvascular changes of retina, neurons, and nephrons and damage of cardiovascular system are a paradigm of this disease, making it one with the highest morbidity and mortality. CHIT1 is elevated and positively intertwined with plasma glucose and age in newly diagnosed, untreated and uncomplicated patients [55,62]. YKL-40 is also found to be elevated in DM2 patients and to positively correlate to insulin resistance and signs of dyslipidemia [63].

Increased production of YKL-40 is an indicator of liver damage. Consequently, in alcoholic liver disease, the level of YKL-40 patients’ plasma positively correlates with the degree of fibrosis [57]. It is also increased in liver cirrhosis caused by the infection with hepatitis-C virus and is suggested as a marker for assessing the level of liver fibrosis and efficacy of anti-HCV treatment [64].

Overexpression of CHIT1 mRNA has been observed in NASH [65]. It was also evidenced that the excessive production of this enzyme leads to the activation of stellate cells, which are a major cell type involved in liver fibrosis [57].

### 3.4. Autoinflammatory Disorders

Both YKL-40 and YKL-39 serve as markers of chondrocyte activation in humans; the latter, however, is more accurate, that is, mRNA of YKL-39 is significantly increased in the cartilage of osteoarthritis patients in comparison to in healthy volunteers. Elevated mRNA expression corresponds also with collagen 2 upregulation. Human chondrocytes express both YKL-40 and YKL-39 mRNA; however, YKL-40 gets downregulated as OA progresses, while YKL-39 is upregulated both in the early and late stages of the disease [57,66].

In rheumatoid arthritis, elevated YKL-40 levels are present. What is more, YKL-40 acts as an autoantigen. Its local release in the joint causes a secondary increase of YKL-40 level in serum, which reflects a degree of the synovial inflammation and joint disruption [57].

### 3.5. Gastric Inflammation

In the study by Cozzarini et al. [14], performed on human gastric biopsies, CHIT1 and AMCase gene expression levels were quantified in order to establish their functions in patients with gastritis associated or not with *Helicobacter pylori (H. pylori)* Notably, the correlation of mRNA expression level of CHIT1, but not AMCase, with *H. pylori* was significant. It was concluded that CHIT1 mRNA is present in the gastric mucosa and it participates in the human immune response to inflammation in general and to *H. pylori* in particular.

## 4. Pathophysiology of Inflammatory Bowel Diseases (IBDs)

IBDs, including CD and ulcerative colitis (UC), are a group of chronic inflammatory disorders predominantly affecting the gastrointestinal (GI) tract [67,68]. They are most prevalent in developing countries that acquire the Western lifestyle, and their incidence is constantly rising [69].

In the CD setting, transmural, diffused inflammation can affect the whole GI tract from the mouth to the rectum. In contrast, UC inflammation is limited only to the mucosa of the colon and the rectum [69]. Despite ongoing studies, the pathogenesis of IBDs still remains unclear [68,69]. It has been widely accepted that dysregulated immune responses, microbial interactions, environmental and genetic factors, and dysfunction in the mucosal barrier are involved [67,68,69,70].

Autoimmune responses lead to inflammation in IBDs. During its acute phase, neutrophils migrate to the inflammation site to serve as a first-line response. Defective neutrophil response regulation in IBDs damages the intestinal barrier. Neutrophils infiltrate the inflamed mucosa and pathogens, such as bacteria that are not phagocytosed and accumulate in the mucosa [69,71]. In addition, phagocytosis and production of reactive oxygen species by neutrophils are defective in IBDs. That leads to continuous recruitment of neutrophils to inflamed mucosa and constant bacterial accumulation [69].

Anti-neutrophil cytoplasmatic antibodies (ANCA) and perinuclear ANCA (pANCA) are present in 10% CD and 70% UC patients. What is more, auto-antibodies to neutrophil proteinase 3 have also been detected in UC. Therefore, the loss of tolerance to neutrophil antigens may play a role in IBD pathophysiology [69,72].

Neutrophils aver innate immune responses, whereas acquired immune responses are provided by immunoglobin A (IgA), which takes part in the defense of intestinal epithelium, protecting the intestinal epithelium from both pathogens and enteric toxins in a noninflammatory manner [69].

Development of IBDs is also dependent on bacteria, especially commensals, and dysregulated host/microbial interactions [67,73]. Commensal bacterial adhesion to intestinal epithelium cells (IECs) and invasion into the host’s IECs are increased. What is more, during the inflammation, general microbial composition in the gut is changed in IBDs. Aggressive, harmful bacteria populations are increased, including adherent invasive *Escherichia coli* (AIEC), *Eubacterium*, and *Peptostreptococcus* sp., *Fusobacterium varium*, *Vibrio cholerae*, *Listeria monocytogenes*, *Bacteroides* and *Helicobacter* species. Of those, especially AIEC and *Bacteroides* species have been found strongly associated with intestinal inflammation in IBDs, especially CD [55,74]. *Escherichia coli* (*E. coli*) antigens and DNA have been detected in granulomatous and peri-ulcerative lesions in CD, and antibodies against the porin protein C of *E. coli* are present in patients with a severe presentation of the disease. Finally, terminal colon of CD patients is often colonized by AIEC possessing the ability to survive extensively within IECs and macrophages without causing apoptosis [75].

## 5. Chitinases in IBDs

Chitinases and chitin lectins are present in the normal human GI tract and participate in immune reactions. Whether remnants of chitin affect the pathogenesis of IBDs is still unclear, but it has been proven that subjects presenting low chitinases levels are more prone to IBDs [76].

### 5.1. CHIT1

CHIT1 production seems to be an important factor in colon homeostasis, since it digests chitin-containing pathogens. Of note, CHIT1 is found to be actively secreted by mucin-producing cells in healthy volunteers and downregulated in patients with active UC. Consequently, decreased secretion of the enzyme seems to cause or worsen the colonic inflammation [55].

### 5.2. AMCase

AMCase is strongly expressed in macrophages and epithelial cells [55]; it is also very abundant in the GI tract and in the lung [57,76]. Moreover, AMCase is upregulated in the Th2-inflammation environment. AMCase-deficient mice show a profound defect in type 2 inflammation after infection with chitin-containing GI nematodes. This leads to impaired immunity that is associated with reduced mucus production and decreased intestinal expression of type 2 response genes, as well as reduced CD103+ dendritic cells in mesenteric lymph nodes [77].

### 5.3. YKL-40

**Figure 1:**
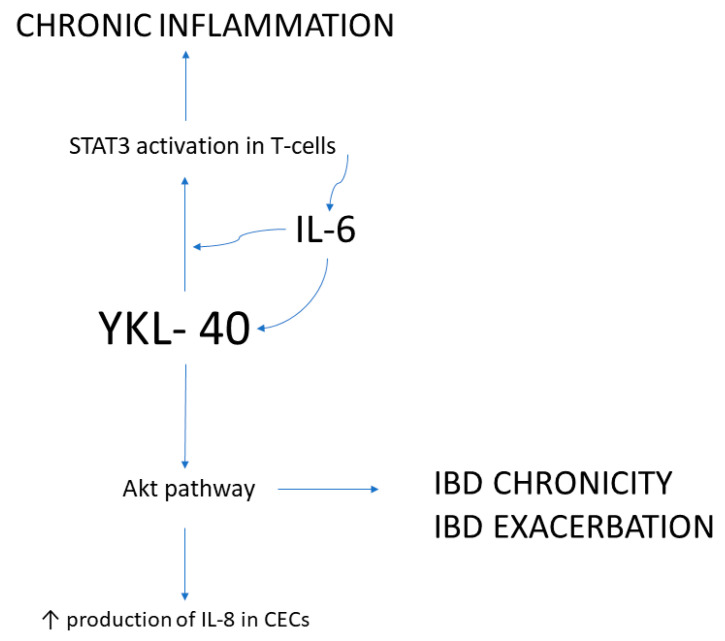
YKL-40 in inflammatory bowel diseases (IBDs).

YKL-40 is produced by epithelial colonic cells, macrophages, and neutrophils, strongly expressed in inflamed mucosa of the colon and overproduced in inflammatory and malignant GI conditions (Figure 1) [76,78].

In IBDs, YKL-40 levels may serve as a marker of the disease severity. In healthy controls, both serum and fecal levels of YKL-40 are almost undetectable. In inflammatory circumstances, however, its expression is vital for a Th2-mediated immune response, as it stimulates the activation of dendritic cells and accumulation of macrophages in the site of inflammation [79]. It acts on STAT3, AKT, and intracellular TLR4 pathways [55]. In vitro experiments using SW480 human colonic epithelial cell lines showed that YKL-40 expression is potently induced by proinflammatory cytokines, i.e., IL-1β, IL-6, and tumor necrosis factor (TNF) [35].

YKL-40 in mice is called the “breast regression protein” BRP-39. In mouse models of dextran sulfate sodium (DSS) -induced colitis, a significant upregulation in BRP-39 gene expression has been found. BRP-39 enhances the adhesion and internalization of intracellular bacteria into colonic epithelial cells. In vivo neutralization of BRP-39 vastly suppresses the development of DSS-induced colitis by decreasing bacterial adhesion and internalization into colonic mucosa and eventually suppresses their translocation into mesenteric lymph nodes, liver, and spleen [35,70].

Other studies on mice showed that BRP-39-deficient mice are more resistant to *Salmonella typhimurium* (*S. typhipharum*) and *AIEC* than non-BRP-39-deficient mice [31,80]. Both bacteria strains are found to induce severe intestinal inflammation in wild-type, but not in BRP-39 knock-out mice. Bacteria-infected BRP-39 knock-out animals present decreased cellular infiltration, bacterial translocation, and production of IL-6 and IL-22. Moreover, they also show aberrant STAT3 activation after bacterial infection [31,80]. What is more, anti-BRP-39 antibody-treated mice present a significantly lower level of *S. typhimurium* in peripheral organs in comparison to in control rabbit-IgG-treated mice [70].

As mentioned above, commensals play an important role in GI immunity. Disrupted colonic microbial balance may lead to increased susceptibility to infection and aberrant immune processes. A good example of this process is infectious enterocolitis, in which physiological colonic flora allows the overgrowth of pathogenic microbes [55]. Inflamed colonic mucosa overexpresses YKL-40, which promotes bacterial adhesion to epithelial cells. YKL-40 is mainly expressed by epithelial cells and macrophages [31,70].

Traditionally, the activity of IBDs is assessed by clinical symptoms and laboratory results, i.e., leukocyte count, serum C-reactive protein levels or erythrocyte sedimentation rate. Those outcomes, however, are not specific to these entities, since they are not produced locally in inflamed sites, but instead in the liver, and do not always mirror the disease activity [81,82]. Feces are useful in evaluating the status of colonic inflammation, that is, fecal calprotectin released from activated neutrophils is a good marker of inflammation of colorectal mucosa, especially since its levels correlate to the severity of inflammation process [83]. The most objective and accurate way to measure the colonic inflammation is colonoscopy, the “gold standard” in IBDs. However, this measure is not suitable for frequent examinations, especially in the pediatric population, as it is invasive, painful and labor-intensive [84].

In general, serum YKL-40 levels correlate with the disease activity of IBD patients. In a study performed by Deutschmann et al., CD patients showed much higher expression of IgG to YKL-40 than UC and celiac disease (CeD) patients [68]. The ratios of IgA and sIgA to YKL-40 were also significantly higher in CD than in UC, CeD, and healthy patients and demonstrated higher prevalence in CD than in healthy controls. It is also associated with a more complicated progression of the disease [69]. In another study by Vind et al., more than half of CD patients presented increased YKL-40 serum levels which, interestingly, did not decrease when the disease became inactive. In UC, half of the patients also presented higher YKL-40 levels, which dropped once the disease became inactive and correlated with disease activity measured by simple clinical activity index (SCCAI) [82,85].

What is more, fecal YKL-40 correlates with the endoscopic activity of both CD and UC. In a study by Atomasu et al., fecal YKL-40 levels were significantly increased in active UC and CD compared to in healthy controls [77]. The levels of fecal YKL-40 were also elevated in patients with intestinal wall thickening in comparison to in healthy patients and those with no small intestine wall thickening [84].

One of the hallmarks of IBDs is the infiltration of mononuclear cells and neutrophils and ulceration of the mucosa, sometimes leading to remodeling of an extracellular matrix (ECM). Clinically fibrosis, a nonspecific result of the chronic inflammation, is seen in both CD and UC [86]. In UC, increased submucosal collagen is observed, whereas in CD inflammatory cells infiltration leads to obstruction and transmural fibrostenosis. YKL-40 has a growth factor activity for fibroblasts, synovial cells and chondrocytes and acts as a chemoattractant for endothelial cells stimulating their migration [86]. It co-works with insulin-like growth factor 1 (IGF-1) in stimulating the fibroblasts growth. Vind et al. in their study on IBD patients found out that patients with active UC had higher serum YKL-40 levels than those with inactive disease and healthy controls while inactive UC patients presented normal serum levels of YKL-40 [81]. It is unknown, however, if YKL-40 is a more sensitive marker than CRP, but YKL-40 is produced locally in the site of inflammation, whereas CRP is produced in the liver in response to high IL-6 production. In the same study, no relationship was found in CD patients between serum YKL-40 and disease activity; 46% of active CD patients and 30% of patients with a mild or quiescent form of the disease presented increased YKL-40 serum levels compared to controls, suggesting YKL-40 elevation also in patients with clinically inactive disease [82]. On the other hand, YKL-40 in CD seems to reflect the ongoing fibrogenesis, as serum YKL-40 levels are closely connected to the level of liver fibrosis and immunohistochemical analysis has shown YKL-40 production in liver fibrosis areas [28].

Rheumatic symptoms are the most frequent extra-intestinal symptoms in IBDs and occur in 5%–20% in IBD patients. Bernadi et al. [87] described that the concentrations of YKL-40 in serum and synovial fluid are closely connected in patients with joint disease, implying that most of the protein detected in serum may be produced within the joint. Increased serum YKL-40 levels in IBDs with joint involvement compared to in IBDs and the absence of influence by bowel inflammation implies YKL-40 is a possible marker of articular destruction in IBDs, especially in comparison to CRP levels, which lack specificity for joint diseases [87]. In a similar study by Gaballa et al. [88], it was confirmed that serum levels of YKL-40 are higher in patients with articular involvement than in those without articular involvement.

Patients suffering from chronic IBDs have been found to have an increased risk (0.5–1%) of developing colitis-associated cancer 10 years from the initial diagnosis, and to-date, a lot of work has been conducted to improve colonoscopic techniques and find a non-invasive serological marker [67]. In UC, colonic YKL-40 mRNA expression is 20-folds elevated in patients with remote neoplastic lesions compared to in healthy controls, suggesting a major or direct role of this protein in inflammation-associated neoplastic transformation. Expression of YKL-40 is found in Lgr5+ stem-like cells, Paneth cells, and neuroendocrine-type cells in UC patients with dysplasia. In contrast, YKL-40-expressing cells are not observed in UC patients without dysplasia [89]. CD patients present a 5.6-fold increase in colonic adenocarcinoma development risk, indicating a possible role of YKL-40 in the initiation or progression of the neoplastic process. Recently, it has been found to be elevated in the visceral fat biopsies in colon cancer patients, which suggests its production not only at the sites of inflammation, but also by visceral fat [90].

IL-6 is a critical tumor promoter in the early stages of colitis-associated carcinoma, because it suppresses apoptosis and reinforces proliferation. IL-6 stimulation enhances YKL-40 production; therefore, blocking IL-6-mediated production of YKL-40 could help prevent inflammation and subsequent colitis-mediated carcinogenesis in epithelial cells [67,91].

## 6. A Brief Review of Human Drug-Like Chitinase Inhibitors

**Figure 2:**
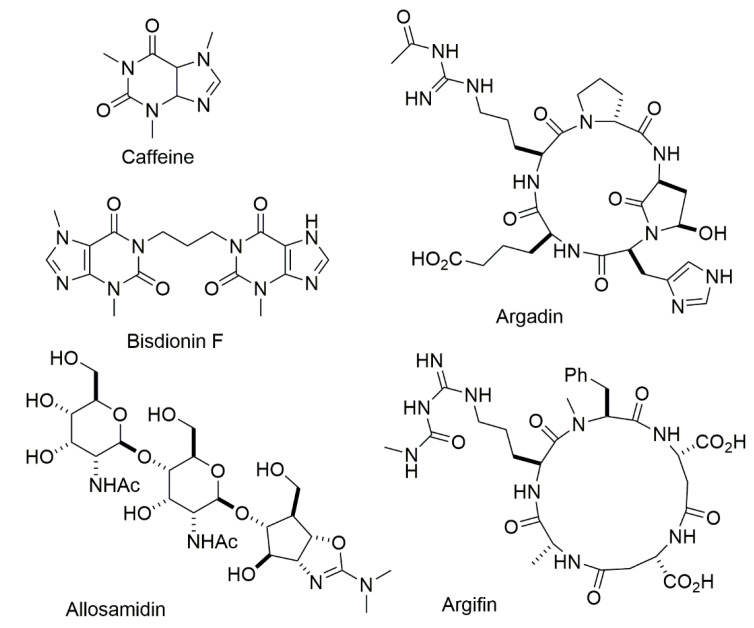
Examples of naturally occurring chitinase inhibitors.

Several classes of naturally occurring chitinase inhibitors have been identified so far. These include pseudosaccharides (i.e., allosamidin and demethylallosamidin) [92], the cyclic peptides (i.e., argadin and argifin) [93] and several xanthine derivatives (i.e., theophylline, caffeine and pentoxifylline) (Figure 2) [94].

**Figure 3:**
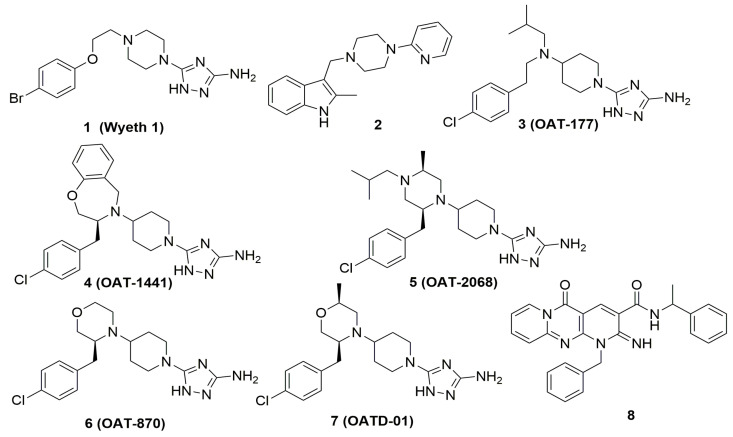
Examples of drug-like chitinase inhibitors.

Xanthine derivatives (theophylline, caffeine, and pentoxifylline) are the first reported drug-like inhibitors of human chitinases. They are identified by screening a commercially available library of drug molecules [94]. These compounds with 1,3-dimethylxanthine substructure have low activity and are not selective chitinase inhibitors. However, their modifications result in the synthesis of a more potent analog, bisdionin C with low micromolar inhibition of both AMCase and CHIT1 enzymes (human CHIT1 (hCHIT1) IC_50_: 8.3 µM; human AMCase (hAMCase) IC_50_: 3.4 µM). A selective inhibitor bisdionin F (hAMCase IC_50_: 0.92 µM; 20-fold selectivity over hCHIT1) was discovered after the analysis of the co-crystal structure of a complex consisting of bisdionin C and AMCase [95]. The Wyeth research group used a combination of high-throughput screening, fragment-based drug design and in silico screening to identify several drug-like inhibitors of AMCase and CHIT1, represented by compounds 1 and 2 [96]. Satisfactory drug-like profile and analysis of crystallographic data of Wyeth 1 compound (PDB code 3RM4) led to the identification of key interactions in the active site of AMCase and hence allowed optimizing inhibitory activity by shifting the position of central nitrogen atom from piperazine to 4-aminopiperidine. This observation led to the discovery of a series of highly potent and selective chitinase inhibitors with aminotriazoles moiety by OncoArendi Therapeutics researchers. Selective mAMCase inhibitor 3 (OAT-177; mAMCase IC_50_: 19 nM) shows a significant anti-inflammatory efficacy in acute house dust mite (HDM)-induced allergic airway inflammation (Figure 3) [97].

As a continuation of this work, a selective hAMCase inhibitor 4 (OAT-1441; hAMCase IC_50_: 7 nM; 177-fold selectivity over hCHIT1) characterized also by low affinity towards hERG ion channel and favorable pharmacokinetic (PK) profile in rodents was also identified [98].

Further investigation of the aminotriazole series leads to the discovery of a highly active and selective mouse CHIT1 inhibitor 5 (OAT-2068; mCHIT1 IC_50_: 29 nM) with good PK properties [99]. These selective compounds can serve as valuable tools in future studies on the roles of AMCase and CHIT1 in in vivo models of various chitinases-implicated pathologies.

The exploration of published X-ray structures of complexes of hCHIT1 and hAMCase with their small-molecule inhibitors and structure–activity relationships analysis within the series of aminotriazoles is an inspiration for the design of dual chitinase inhibitors [100]. Out of those, compound 6 (OAT-870; human/ mouse (h/m) CHIT1 IC_50_: 48/74 nM; h/mAMCase IC_50_: 22/30 nM) represents an advanced leading compound with an optimized in vitro pharmacological profile in terms of dual inhibition of chitinases against both human and murine AMCase and CHIT1 enzymes. Compound 6 is also characterized by a very good PK profile in mice and rats and shows a significant reduction of HDM-induced pulmonary inflammation in mice. In addition, affinity toward the hERG potassium ion channel of compound 6 is significantly reduced when compared to the earlier reported chitinase inhibitors. However, its relatively high activity against dopamine transporter (DAT IC_50_: 370 nM) is considered to be a serious liability prohibiting further development of this molecule. This investigation leads to the identification of compound 7 (OATD-01; h/mCHIT1 IC_50_: 23/28 nM; h/mAMCase IC_50_: 9/7.8 nM)—a clinical candidate that currently completes phase 1b of the clinical trials. OATD-01 shows anti-fibrotic efficacy in the bleomycin-induced murine model of pulmonary fibrosis and can serve as a new therapy against IPF and possibly other fibrosis-related diseases [101].

Recently, Jiang et al. used a structure-based virtual screening to identify a series of chitinase inhibitors with a common dipyrido-pyrimidine scaffold [102]. The most promising candidate is an 80-fold selective hCHIT1 inhibitor compound 8 with a Ki value of 49 nM, however, in murine enzymes; this molecule was only 15-fold more selective for mCHIT1 compared to mAMCase. The compound was further evaluated in a selected set of key enzymes, transporters and ion channels to characterize its safety. Compound 8 showed potential cardiotoxicity (98.8% inhibition at 10 μM for hERG) and revealed inhibition of phosphodiesterase PDE4D2 (87.8% inhibition at 10 μM).

The in vivo efficacy of compound 8 was evaluated in a murine model of bleomycin-induced lung fibrosis. Compound 8 showed a significant reduction of lung fibrosis but also increased lung inflammation, which may suggest a protective role of chitinases. This result is opposite to the data published by OncoArendi Therapeutics.

No selective drug-like small molecules binding to YKL-40 have been identified so far.

## 7. Chitinase Inhibitors in IBDs and Colorectal Cancer Models

Several xanthine derivatives were reported to be tested in animal models of various human GI tract pathologies, such as IBDs and colorectal cancer.

Theophylline, the main component of the purine alkaloids found in tea plants, is identified as a pan-family 18 chitinase inhibitor. Research by Johansen et al. [103] revealed the presence of high serum levels of YKL-40 in patients with rectal cancer. Chen et al. confirmed that YKL-40 is highly expressed in the SW480 cell line and that treatment with exogenous YKL-40 could significantly promote SW480 proliferation and migration [89,104]. These findings support the hypothesis that YKL-40 serves a key role in inflammation-associated neoplastic changes.

Peng et al. [105] revealed that the expression level of YKL-40 is decreased in theophylline-treated SW480 cell lines. The proliferation of SW480 cells is suppressed following incubation with theophylline, which is associated with G1 phase cell cycle arrest and a decrease in the expression of angiopoietin-2. The mechanism of theophylline action may involve the downregulation of YKL-40 expression, the arrest of the cell cycle at G1 phase and the inhibition of angiopoietin-2 expression. These results support the potential use of anti-YKL-40 and anti-angiogenic strategies in the treatment of rectal cancer.

In vitro treatment of IECs by caffeine (1,3,7-trimethylxanthine as a pan-chitinase inhibitor) decreases YKL-40 mRNA expression, which results in reduced bacterial invasion in a dose-dependent manner [73]. In vivo, mice treated with caffeine show a delayed response to DSS-induced colitis, reduced bacterial translocation into other organs and decreased cytokine production. Caffeine treatment also causes a downregulation of YKL-40 and a significant suppression-associated AKT signaling pathway (both in vivo and in vitro). The availability of caffeine in many natural sources including foods and beverages and its mucosal protective effect with minor adverse effects on the cardio-respiratory system make it a good candidate to target chitinase-mediated IBD pathogenesis [73,75,106].

In addition, pentoxifylline was tested in animal models of IBDs. Peterson et al. [107] reported that intra-rectal administration of pentoxifylline or its metabolite lisofylline in a murine 2,4,6-trinitrobenzenesulfonic acid (TNBS)-induced colitis model alleviates colonic inflammation and intestinal fibrosis. Another study demonstrated similar improvement by pentoxifylline in TNBS-colitis in rats [108]. Interestingly, Murthy et al. showed that combining pentoxifylline with anti-TNF antibody in DSS-induced colitis mice can reduce side effects associated with anti-TNF antibody treatment alone [109]. Ex vivo studies also showed that peripheral mononuclear cells, obtained from the inflamed mucosa of CD and UC patients, reduce TNF secretion by 50% in the presence of pentoxifylline (up to 100 μg/mL) for 24 h [110].

## 8. Conclusions and Perspectives

There is a growing body of evidence for the association of chitinase expression and activity in the pathophysiology of IBDs. As evidenced by the literature, the strongest focus is on YKL-40 due to its diagnostic and therapeutic potential. Serum and fecal YKL-40 levels that reflect the intensity of inflammation could serve as a tool to monitor the disease activity [55]. Moreover, fecal YKL-40 assays may be useful in predicting the severity and activity of mucosal inflammation in IBDs [84]. The discovery that methylxanthine derivatives, including caffeine, theophylline, and pentoxifylline, can potentially suppress inflammation via their interaction with YKL-40 (or with BRP-39 in animal models) is also of great importance. However, it must be stressed that this interaction has been confirmed only indirectly by the measurement of the corresponding mRNA expression level. While it might be interesting to investigate how the methylxanthines bind to YKL-40 and BRP-39 proteins by the microscale thermophoresis and AlphaScreen assays [111], the promiscuous nature of the biological activity of methylxanthines disqualify them as potential drugs. Therefore, the development of novel and selective small-molecule drugs that would inhibit YKL-40 activity appears to be an appealing approach to refractory IBD therapy. Ongoing research in this field is of great necessity and is strongly warranted.

## Data Availability

Not applicable.
